# Characterization of Novel Lytic Bacteriophages of *Achromobacter marplantensis* Isolated from a Pneumonia Patient

**DOI:** 10.3390/v12101138

**Published:** 2020-10-08

**Authors:** Hiu Tat Chan, Heng Ku, Ying Ping Low, Steven Batinovic, Mwila Kabwe, Steve Petrovski, Joseph Tucci

**Affiliations:** 1 Department of Physiology, Anatomy and Microbiology, La Trobe University, Bundoora, Victoria 3083, Australia; m.chan@latrobe.edu.au (H.T.C.); S.Batinovic@latrobe.edu.au (S.B.); steve.petrovski@latrobe.edu.au (S.P.); 2 Department of Microbiology, Royal Melbourne Hospital, Victoria 3000, Australia; iphyll66@yahoo.com; 3 Department of Pharmacy and Biomedical Science, La Trobe Institute for Molecular Science, La Trobe University, Bendigo, Victoria 3552, Australia; H.Ku@latrobe.edu.au (H.K.); M.Kabwe@latrobe.edu.au (M.K.)

**Keywords:** bacteriophage, *Achromobacter marplatensis*, cystic fibrosis, pneumonia

## Abstract

*Achromobacter* spp. are becoming increasingly associated with lung infections in patients suffering from cystic fibrosis (CF). *A. marplatensis*, which is closely related to *A. xylosoxidans*, has been isolated from the lungs of CF patients and other human infections. This article describes the isolation, morphology and characterization of two lytic bacteriophages specific for an *A. marplatensis* strain isolated from a pneumonia patient. This host strain was the causal agent of hospital acquired pneumonia–the first clinical report of such an occurrence. Full genome sequencing revealed bacteriophage genomes ranging in size from 45901 to 46,328 bp. Transmission electron microscopy revealed that the two bacteriophages AMA1 and AMA2 belonged to the *Siphoviridae* family. Host range analysis showed that their host range did not extend to *A. xylosoxidans*. The possibility exists for future testing of such bacteriophages in the control of *Achromobacter* infections such as those seen in CF and other infections of the lungs. The incidence of antibiotic resistance in this genus highlights the importance of seeking adjuncts and alternatives in CF and other lung infections.

## 1. Introduction

*Achromobacter* spp. are emerging pathogens in cystic fibrosis (CF) patients, as well as immunocompromised and immunocompetent patients [1,2]. They can cause pulmonary infections, bacteremia, cellulitis, ophthalmic infections and meningitis [3,4,5,6]. In CF patients, *Achromobacter* infections are associated with pulmonary exacerbation, accelerated decline in lung function, need for lung transplantation and death [7,8,9]. Clinically, *A. xylosoxidans* is the most frequently isolated in this genus, and therefore the most studied. However, with application and improvement of genetic identification techniques, it is now apparent that other *Achromobacter* spp. are also important in human health. Furthermore, these species are commonly erroneously misidentified as *A. xylosoxidans* [10,11,12,13,14].

*A. marplatensis,* which is phylogenetically close to *A. xylosoxidans*, was originally isolated from soil with few reports of human infection. The recent review of genetic divergence within the *Achromobacter* genus resulted in an increase in the number of recorded strains of *A. marplatensis*, owing to the reclassification of *A. spiritinus* as *A. marplatensis* [15,16]. *A. marplatensis* now carries a newly recognised importance in CF patients, being isolated from more than 10% of patients in a French CF Centre [12], as well as in CF patients in Argentina [17], Serbia [18], Denmark [19] and the UK [20].

*A. marplatensis* carries antibiotic resistance mechanisms and can exhibit high minimum inhibitory concentrations for a range of common antibiotics [17]. This, together with the absence of clinical breakpoints (referring to the categorization of a micro-organism as clinically susceptible, clinically intermediate or clinically resistant based upon response to clearly specified concentrations of an antimicrobial agent) for *Achromobacter* in guidelines published by the Clinical and Laboratory Standards Institute and European Committee on Antimicrobial Susceptibility Testing, may complicate the management of *A. marplatensis* infections. Environmentally, *A. marplatensis* can metabolise important organic pollutants such as pentachlorophenol, an environmental toxin which very few micro-organisms are capable of degrading [16,21]. Therefore, it is an important organism that can cause life threatening infections, and one that potentially could be utilised for environmental bioremediation.

Bacteriophages are an important natural predator against bacteria in the ecosystem, and may be utilised to treat human infections [22,23] as well as to modulate the microbiota during bioremediation in a range of environments including wastewater, activated sludge and contaminated soil [24,25,26]. There have been published reports of bacteriophages lytic against *A. xylosoxidans* [27,28,29,30], including their use in clinical therapy against *A. xylosoxidans* lung infection in CF patients [31]. To date, there have been no reports of bacteriophages lytic against *A. marplatensis*. This work describes the isolation, purification, morphological and genomic characterisation of novel bacteriophages AMA1 and AMA2. These bacteriophages are lytic against an *A. marplatensis* strain that caused hospital acquired pneumonia in a trauma patient. To the best of our knowledge, this is the first reported case of pneumonia caused by *A. marplatensis*, and so the isolation of these bacteriophages is important in that it opens up the potential, in future, for complementation of antibiotic therapy in CF and other respiratory infections caused by *A. marplatensis*.

## 2. Materials and Methods

### 2.1. Ethics Approval

All methods were performed in accordance with the La Trobe University Ethics, Biosafety and Integrity guidelines and regulations. Clinical isolates of *Achromobacter* were obtained from specimen cultures as part of routine care. *A marplatensis* was isolated from the sputum of a trauma patient suffering from hospital acquired pneumonia. The study protocols were approved by the La Trobe University Ethics Committee, reference number: S17-111 (Ethical permission was granted on 7 March 2017).

### 2.2. Bacterial Cultures and Identification

All strains were grown using nutrient agar or tryptone soya broth (TSB) and agar (Oxoid, Adelaide, Australia), at 37 °C under aerobic conditions. Preliminary genus identification was performed by matrix assisted laser desorption ionisation–time of flight mass spectrometry (MALDI-TOF, Bruker Daltonik, Germany). Sequencing of the 16s rRNA gene and the *nrdA* housekeeping gene was also used to assist identification. PCR was performed using rRNA gene universal primers 27F, 519R, 895F and 1492R [32,33,34] and *nrdA* gene primers P1, P2 [11,12] and the products were cleaned with an Ultra Clean® DNA purification kit (MO-BIO, California, USA) before Sanger sequencing at the Australian Genome Research Facility (Brisbane, Australia). The *nrdA* sequences were submitted to the MLST *Achromobacter* database (https://pubmlst.org/achromobacter/) for analysis.

### 2.3. Isolation and Purification of Bacteriophages

Samples of garden soil and castings from composting worms from Brunswick West, Victoria, Australia were used to screen for bacteriophages. Briefly, bacterial lawns of *A. marplatensis* on nutrient agar (Thermofisher, Australia) were prepared by using a cotton swab. The soil and worm casting samples were first incubated at room temperature for 2 d with 15 mL of cooked meat media (Oxoid, Adelaide, Australia) with constant agitation, then centrifuged (17,000× *g* for 5 min) and filtered through 0.2 µM pore size cellulose acetate filters (Advantec, Melbourne, Australia). The filtrate (10 μL) was placed onto the bacterial lawn and plates were incubated for 24 h. Any potential bacteriophage clearing was excised (along with a portion of agar) and resuspended in 500 μL of TSB, before centrifugation (17,000× *g* for 5 min) and a 10-fold serial dilution was completed. A total of 10 μL of each dilution was placed on a bacterial lawn such that plaques could be observed. This serial dilution purification was repeated five times to ensure single virion infection. 

### 2.4. Extraction of Bacteriophage DNA

Five mL of phage stock (>10^9^ PFU/mL) was used to extract bacteriophage DNA and all chemicals used here were from Sigma (Sydney, Australia) unless stated otherwise. Extraneous DNA present in the phage stock was digested at room temperature using 10 μg/mL DNase I and RNase A and 5 mmol/L MgCl_2_, for 30 min. The bacteriophages were recovered by overnight polyethylene glycol (PEG) precipitation using 10% (*w*/*v*) PEG 8000 and 1 mol/L sodium chloride at 4 °C. The precipitated bacteriophages were centrifuged (17,000× *g* for 15 min) and resuspended in nuclease free water (Promega, Sydney, Australia). Bacteriophage proteins were digested for one hour at 55 °C with the addition of 50 μg/mL Proteinase K, 20 mmol/L EDTA and 0.5% (*v*/*v*) sodium dodecyl sulphate. After the incubation, an equal volume of phenol–chloroform–isoamyl alcohol (29:28:1) was added and centrifuged (17,000× *g* for 5 min). The aqueous phase was carefully removed before the addition of an equal volume of isopropanol. The DNA was precipitated overnight at −20 °C, then collected by centrifugation (17,000× *g* for 10 min). The DNA pellet was washed with 70% ethanol, air dried and resuspended in 25 μL of nuclease free water (Promega).

### 2.5. Host Range Analysis

To assess for lytic capacity, bacteriophage stocks (>10^7^ PFU/mL) were diluted 10-fold serially and tested on an *A. xylosoxidans* strain. A bacterial lawn of the strain was prepared and 10 μL of each dilution of each stock was placed on the lawn. If individual plaques were observed, it was noted that the bacteriophage could target the strain.

### 2.6. One Step Growth Analysis

The methods for the one step growth analysis were similar to those described previously [35]. *A. marplatensis* was grown to exponential phase in TSB, and collected by centrifugation at 12,000× *g* for 5 min. Bacterial cells were resuspended in fresh TSB at a concentration of 0.6U (OD_600_). A total of 100 µL of AMA1 and AMA2 bacteriophage stocks of approximately 10^7^ PFU/mL were added to separate tubes with 900 μL of the *A. marplatensis* cells, at a multiplicity of infection (MOI) of 0.01, and incubated at 4 °C for 15 min to allow for adsorption. Adsorbed bacteriophages were collected by centrifugation at 12,000× *g* for 5 mins and resuspended in 50 mL of fresh TSB. The mixture was incubated aerobically at 37 °C. One mL samples were taken by centrifugation at 12,000× *g* for 2 min every 10 min to calculate viable bacteriophage concentrations (PFU/mL) [36].

### 2.7. Transmission Electron Microscopy

Electron microscopy was performed using 400-mesh formvar and carbon copper grids (ProSciTech, Townsville, Australia). Bacteriophage stocks (>10^7^ PFU/mL) were allowed to adsorb to the grid for one minute before excess solution was removed using filter paper. The grids were then negatively stained three times with 2% (*w*/*v*) uranyl acetate for 20 seconds. Excess stain was removed by filter paper and grids were air-dried for 20 min before examination under a JEOL JEM-2100 transmission electron microscope (TEM). This was operated at an accelerating voltage of 200 kV and high-resolution digital images were recorded on a Gatan Orius SC200D 1 wide angle camera with Gatan Microscopy Suite and Digital Micrograph (Version 2.32.888.0) imaging software. Viruses were measured using GMS3 software.

### 2.8. Genomic Characterization and Phylogeny

Bacteriophage DNA was sequenced using an Illumina MiSeq® next generation sequencing platform. The DNA library was prepared by using a Nextera® XT DNA sample preparation kit according to the manufacturer’s instructions and sequenced using a MiSeq® V2 reagent kit (300 cycles), as 150 bp paired end reads. Reads were assembled de novo within Unicycler v0.4.8 [37]. Open reading frames of these sequences were predicted using Glimmer 3 [38], Prodigal V.2.6.2 [39] and manually checked by Geneious prime V.2019.0.3. Bacteriophage genomes were annotated using Blast+ (Version 2.9.0) nr database with a cut off e-value of (1e-5) [40], NCBI Web CD-search Tool (Pfam database) with a cut off e-value of 0.01 [41] and HHpred (default database:PDB_mmCIF70_23_Jul; Pfam-A_V33.1; NCBI_Conserved_Domain (CD)_v3.18). Proteomic trees were constructed with ViPTreeGen (Version 1.1.2) using sequence similarity distance based on tBLASTx results and construct (bio)nj tree [42]. iTOL was used [43] to annotate the proteomic tree. tRNAs were predicted by ARAGORN [44] and tRNAscan-SE-2.0.5 [45].

### 2.9. Codon Usage Analysis

Geneious prime V.2020.0.3 was employed to extract nucleotide sequences from genome annotations of *A. marplantensis* (NZ_ALJE00000000.1), AMA1 and AMA2. For *A. marplantensis* (NZ_ALJE00000000.1), 177 incomplete ORF annotations were excluded. Biopython script was used to remove the start codons by slicing nucleotide sequence [3]. *A. marplantensis* (NZ_ALJE00000000.1), AMA1 and AMA2 codon usage was calculated using the Sequence Manipulation Suite of JavaScript programs for analyzing and formatting protein and DNA sequences [46]. 

Codon usage analyses to investigate the adaptive role of bacteriophage encoded tRNAs have been described previously [47]. Briefly, for the 64 codons, a relative codon frequency, *fi*, was calculated using the following formula: *f_i_* = *Number of codon_i_/ Number of all codons.* The ratio of relative codon frequencies, *ri*, between bacteriophage and host was then assessed as: *r_i_* = *f_i_^bacteriophage^/f_i_^host^.* If *ri* was ≥ 1.1, the bacteriophage was considered to exhibit a higher relative codon frequency for codon *i* than the host; If *ri* was between 0.9 and 1.1, the bacteriophage and host were considered to have similar relative codon frequencies; if *ri* was < 0.9, the host was considered to exhibit a higher relative codon frequency [47].

## 3. Results

### 3.1. Bacterial Identification

Results from MALDI-TOF analysis suggested that the strains that were to be used in this study were *A. denitrificans* and *A. xylosoxidans*. However, the more sensitive sequencing of the 16s rRNA gene (accession numbers (MW032195; MW032196) revealed the identity of the strains as *A. marplatensis* and *A. xylosoxidans*. Therefore, there was discordance between the MALDI-TOF and genetic analyses in the identification of *A. marplatensis.* Analysis of the *nrdA* housekeeping gene confirmed the identity of the host strain as *A. marplatensis*.

### 3.2. Isolation and Phenotypic Characterization of Novel A. marplatensis Bacteriophages

**Figure 1:**
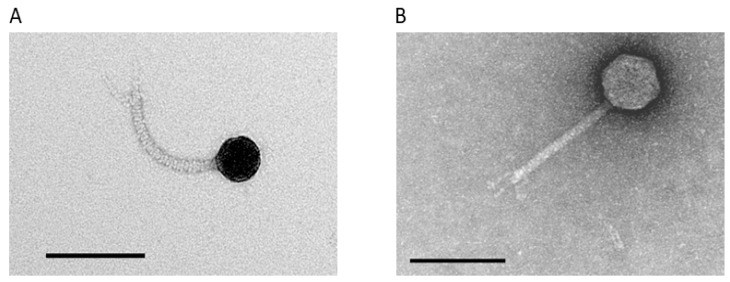
TEM characterization of *A. marplatensis* bacteriophages. (**A**) AMA1: capsid length approx. 43 ± 0 nm; tail length and width approx. 125 ± 2 nm and 10 ± 0 nm, respectively. (**B**) AMA2: capsid length approx. 61 ± 0 nm; tail length and width approx. 120 ± 3 nm & 10 ± 0 nm, respectively. Ten AMA1 and AMA2 virus particles were measured to calculate these sizes (Micron marker = 100 nm).

**Figure 2:**
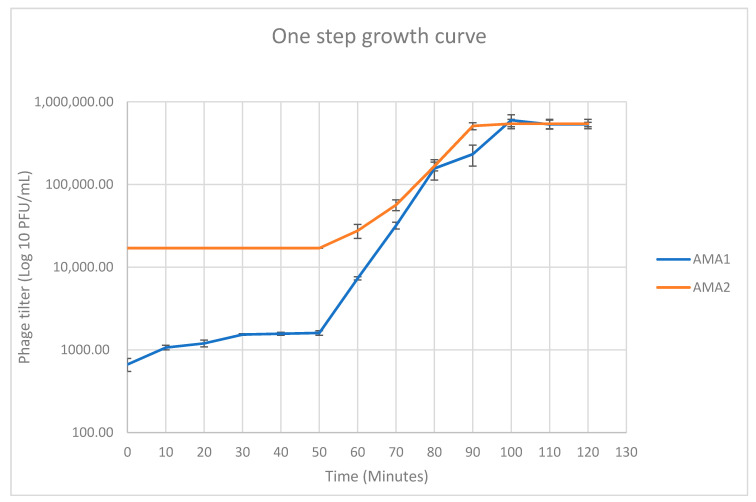
One step growth curve for AMA1 (blue line) and AMA2 (orange line) on *A. marplatensis* clinical strain. Error bars represent standard error of mean, calculated from three independent experiments.

Two bacteriophages were isolated from samples of garden soil and castings from composting worms from Victoria, Australia. Plaques formed by these bacteriophages were approximately 1 mm in diameter, and purified preparations were termed AMA1 and AMA2. Transmission electron microscopy revealed that AMA1 and AMA2 were both of the *Siphovirus* morphotype (Figure 1). AMA1 had a capsid length approx. 43 ± 0 nm; tail length and width approx. 123 ± 2 nm and 10 ± 0 nm, respectively. AMA2 had a capsid length approx. 61 ± 0 nm; tail length and width approx. 120 ± 3 nm and 10 ± 0 nm, respectively (Figure 1). The host range of these bacteriophages was limited to the *A. marplatensis* strain used here; they did not lyse the *A. xylosoxidans* strain. One step growth experiments showed that the latent periods were 50 min for both AMA1 and AMA2 (Figure 2). The burst sizes were 34 ± 5 PFU/infected bacteria for AMA1 and 19 ± 1 PFU/infected bacteria for AMA2. 

### 3.3. Genome Analysis of A. marplatensis Bacteriophages AMA1 and AMA2

**Figure 3:**
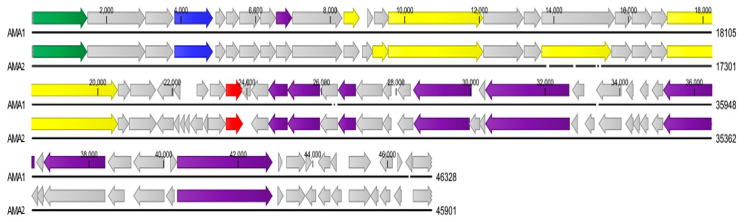
AMA1 and AMA2 genome alignments. The arrows indicate the putative predicted ORFs and direction of transcription. Grey arrows indicate putative hypothetical genes; green arrows indicate putative terminase genes; blue arrows indicate putative capsid genes; yellow arrows indicate putative tail fibre genes; purple arrows indicate putative DNA manipulation and metabolism genes; red arrows indicate putative lysis genes.

The genome sequence data for each bacteriophage were assembled de novo. Four contigs were generated for AMA1: the main contig was 46328 bp with an average coverage of 1123x, contig two was 37476 bp with an average coverage of 27x, contig three was 1369 bp with an average coverage of 8x, contig four was 1094 bp with an average coverage of 7x. One contig was generated for AMA2, with an average coverage of 1382x. The raw sequence data were submitted to the Sequence Read Archive (SRA: PRJNA665751). The AMA1 bacteriophage genome (Accession number: MT241605) had a genome size of 46,328 bp and was composed of 62 predicted ORFs (Table 1; Figure 3), of which 22.5% (14/62) were related to genes encoding putative functional proteins (Table 2). The GC content of AMA1 was 56.3%. The AMA2 bacteriophage genome (Accession number: MT241606) had a genome size of 45901 bp and was composed of 68 predicted ORFs (Table 1; Figure 3), of which 22.1% (15/68) were related to genes encoding putative functional proteins (Table 2). The GC content of AMA2 was 54.5%. The published genome of *A. marplatensis* shows that the GC% content of this bacteria is 65.10% (NZ_ALJE00000000.1). No putative integrase genes or toxin genes were detected in the genomes of AMA1 or AMA2.

The genomes of the bacteriophages were aligned using the CLC genomics workbench. AMA1 contained a genome which displayed 84.05% identity to AMA2 over 65% of their genomes (NCBI BLAST query cover: 65%; percentage identity: 84.05%).

### 3.4. Presence of tRNA Genes in AMA1 and AMA2 Genomes 

One putative tRNA gene sequence was predicted in the genomes of AMA1 and AMA2 (according to both the Aragorn and tRNAscan-SE 2.0v programs). Each of these tRNA genes was 77 bp, and contained the anticodon tgg, corresponding to codon CCA and the amino acid proline. 

### 3.5. Bacteriophage Phylogeny 

**Figure 4:**
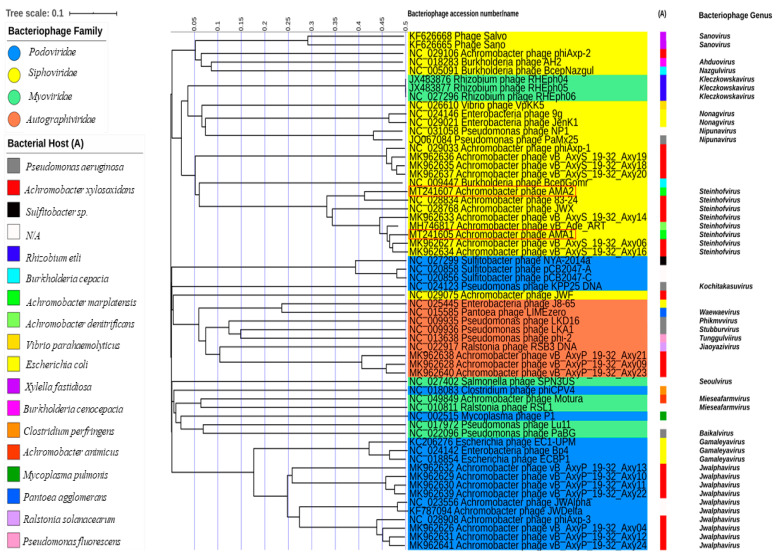
Proteomic tree showing the genome-wide proteomic diversity of published *Achromobacter* and related bacteriophages [28,29,30,48,49,50,51,52,53,54,55,56,57,58,59,60,61,62,63,64,65,66,67,68,69,70,71,72,73]. Red boxes indicate bacteriophages presented here.

Prior to this study there were 26 reported bacteriophages against *Achromobacter* spp. which have had their genomes completely sequenced and fully annotated. The two bacteriophages reported here are the first lytic bacteriophages to be isolated against a clinical strain of *A. marplatensis*. ViPTree online was used to generate a proteomic tree based on genome wide sequence similarities, computed by tBLASTx, for the 26 previously reported *Achromobacter* bacteriophages, those isolated in this study, and 32 bacteriophages that are closely related, including those that infect respiratory pathogens. AMA1 and AMA2 are part of a monophyletic clade that represents the genus *Steinhofvirus*. The proteomtic tree revealed that AMA2 clustered most closely to *Achromobacter* bacteriophage NC 028834 (which also belongs to the *Steinhofvirus* genus). AMA1 clustered more closely to *Achromobacter Steinhofvirus* bacteriophages MK962627, MK962634 and MH746817. AMA1, AMA2 and these related viruses are all members of the *Siphoviridae* family. Interestingly AMA1, AMA2 and the other *Achromobacter Steinhofvirus* bacteriophages form a clade with bacteriophage NC009447 which targets the CF pathogen *Burkholderia cepacia.* As such, they are more closely related to the bacteriophage that attacks this respiratory pathogen than they are to the other *Achromobacter* bacteriophage *Siphoviruses* (Figure 4). 

### 3.6. Codon Usage Analysis in AMA1, AMA2 and A. marplantensis

**Figure 5:**
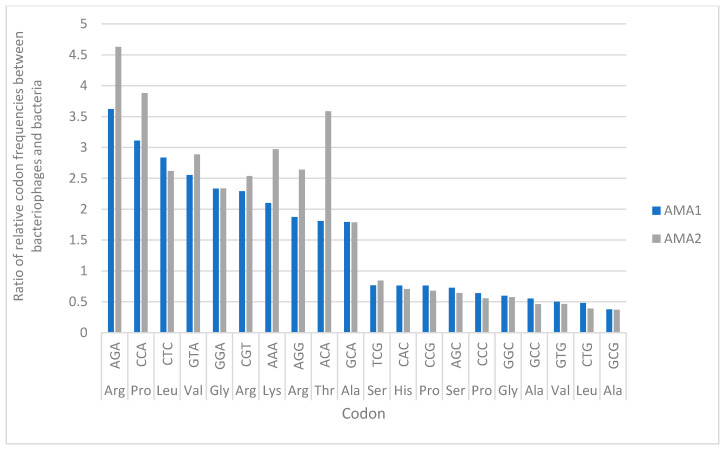
The ratio of relative codon frequencies between bacteriophages AMA1, AMA2 and *A. marplatensis* (NZ_ALJE00000000.1). The figure shows the 10 codons for which the bacteriophages exhibited the highest relative codon frequencies compared to the host, as well as the 10 codons for which the bacteriophages exhibited the lowest relative codon frequencies compared to the host.

Analysis of the relative codon frequencies for AMA1, AMA2 and *A. marplatensis* (NZ_ALJE00000000.1) revealed that the frequency of usage of the CCA codon (generated by the tgg anticodon) was higher in the bacteriophages, compared to their host. The ratio of usage of CCA by the bacteriophages, compared to the *A. marplatensis* bacteria, was the second highest of all the codons (Figure 5).

## 4. Discussion

This is the first report of the isolation and characterisation of bacteriophages lytic against *A. marplatensis*. These bacteriophages were only tested against a single strain, and it is not clear whether they target other strains of this or other bacteria. Yet the importance of this work is highlighted by the fact that the host *A. marplatensis* strain on which these viruses were grown was isolated from a patient suffering from hospital acquired pneumonia. To our knowledge, this is the first report of pneumonia caused by *A. marplatensis.* These bacteriophages AMA1 and AMA2 were *Siphoviridae* viruses with non-contractile tails. They form part of a monophyletic clade that represents the genus *Steinhofvirus*, which clusters more closely to a bacteriophage which targets the CF pathogen *Burkholderia cepacia* than to other *Achromobacter Siphovirus* bacteriophages. Their host range did not extend to *A. xylosoxidans*, highlighting specificity for the host strain. With respect to replication kinetics, their latent period was 50 min, with a burst size of approximately 34 PFU/cell for AMA1 and 19 PFU/cell for AMA2. This compares to other *Achromobacter Steinhofvirus* bacteriophages which have a reported latency of between 55–76 min, and a burst size of approximately 78 PFU/cell [28]. Neither AMA1 or AMA2 possessed any putative integrase genes or toxin genes, which may improve their capacity to be applied in therapy. 

The GC% content of the genomes of these viruses ranged between 54.5 to 56.3% and they each contained a single tRNA gene. It has been previously suggested that more virulent bacteriophages may have a lower GC% composition than their hosts [74]. This was seen for AMA1 and AMA2, as the genome composition of these bacteriophages was significantly different to that of their host bacteria, whose GC% content is 65.1% (NZ_ALJE00000000.1). Each of the bacteriophages we isolated carried a single tRNA gene of 77 bp in length, with the tgg anticodon sequence, corresponding to the codon CCA and the amino acid proline. Analysis of the relative codon frequencies for *A. marplatensis* (NZ_ALJE00000000.1) and bacteriophages AMA1 and AMA2 revealed that the frequency of usage of the CCA codon is higher in the bacteriophages, compared to their host. The ratio of usage of the CCA codon by the bacteriophages, compared to the *A. marplatensis* bacteria, is the second highest of all the codons. Previous suggestions have been for an adaptive role of bacteriophage encoded tRNAs, in that they are selected in bacteriophages to compensate for differences in codon usage between the bacteriophage and the host [75]. This supports the concept that presence of tRNAs in bacteriophages may indicate the requirement for additional translational mechanisms to complement those provided by the host cell. Interestingly, in our experiments, the bacteriophages exhibited the highest frequency of usage for the codon AGA (arginine), relative to the host, yet the tRNA gene with the anticodon sequence for this was not detected in AMA1 or AMA2. The reasons for this are unclear.

Clinically, the identification of *Achromobacter* to species level is difficult, even with the latest MALDI-TOF technologies. The top two matches for our *A. marplatensis* isolate were “*Achromobacter* spp.” and “*Achromobacter denitrificans*” by the MALDI-TOF, and *A. marplatensis* has been previously incorrectly identified as *A. xylosoxidans* in clinical settings [13,17]. In fact, most non-*xylosoxidans* spp. are commonly misidentified as *A. xylosoxidans* in clinical laboratories [13,14]. Differentiation between *A. marplatensis* and *A. xylosoxidans* requires genetic analyses [10,11,12], which in turn require molecular techniques and databases that are not readily available in a routine clinical laboratory. This issue with *Achromobacter* speciation in diagnostic laboratories has two important implications. Firstly, the occurrence and significance of *A. marplatensis* in clinical disease is likely to have been under-estimated. Secondly, when attempting to use bacteriophage cocktails to treat an infection caused by “*A. xylosoxidans*” as identified by a routine clinical laboratory, the cocktail could potentially include bacteriophages against not only *A. xylosoxidans*, but also *A. marplatensis* and other *Achromobacter* spp., because the reported “*A. xylosoxidans*” could in fact be a non-*xyloxisidans* spp. This issue highlights the importance of the adoption of appropriate genetic analyses [11,12,13] for diagnostic laboratories working with *Achromobacter* spp. Such practices would assist in the establishment of reliable *Achromobacter* bacteriophage biobanks which could be assessed in infections caused by the different species of this genus. As such, novel bacteriophages such as AMA1 and AMA2 as reported here, and bacteriophages against other non-*xylosoxidans Achromobacter* spp. could be important constituents of future formulations of bacteriophage therapies for diseases commonly caused by *Achromobacter* spp., such as CF lung infections.

Bacteriophages can be produced in liquid suspensions, stabilised in solid state through lyophilisation, and formulated in liposomal encapsulations, so their delivery via a nebuliser, pressurised metred dose inhaler and as a dry powder inhalation [76] is potentially feasible for treating lung infections in CF. Regulatory bodies such as the European Medicines Agency have suggested that development of bacteriophage therapies should be expedited, but that more evidence based data are required for their approval [77]. While it is hoped that such clinical data can be accumulated in a reasonable time, the application of inhaled bacteriophage therapy to complement antibiotics may provide important options in the prevention and treatment of bacterial infections of the respiratory tract [76].

In conclusion, we report here the isolation, morphological and genomic characterisation of novel bacteriophages lytic for *A. marplatensis*. This bacterium is an important environmental micro-organism capable of breaking down xenobiotics and environmental bioremediation. In humans it is an opportunistic pathogen seen in CF and other lung infections and its resistance to antibiotics means that alternatives such as bacteriophages may provide a useful measure for its control in the future. 

## Figures and Tables

**Table 1:** AMA1 and AMA2 genome data and closest matches.

Bacteriophage	Genome Size	GC Content	Accession Number	Closest Organism Match and Nucleotide Similarity (%)
AMA1	46,328 bp	56.30%	MT241605	*Achromobacter* phage vB_AxyS_19-32_Axy06, (91.3%)
AMA2	46,155 bp	54.50%	MT241606	*Achromobacter* phage 83-24, (79.8%)

**Table 2:** Presence of ORFs in AMA1 and AMA2 whose predicted protein function is known. Predicted function based on Pfam database of conserved functional domains, except for Capsid protein*, whose function was based on HHpred prediction through PDB archive of three-dimensional structures.

Bacteriophage and ORF Number	Putative Protein Product	Pfam
AMA1_49	Autophagy protein Apg6/FlgN protein	Pfam17675
AMA1_5; AMA2_58	Capsid protein*	
AMA1_39; AMA2_26	DNA helicase	Pfam00271
AMA1_41; AMA2_29	DNA polymerase I	Pfam00476
AMA1_53; AMA2_44	DNA primase/polymerase	Pfam09250
AMA1_2; AMA2_55	Terminase large subunit	Pfam04466
AMA1_12;AMA2_7	Tail protein	Pfam13550
AMA1_15; AMA2_68	Tail length tape-measure protein	Pfam09718
AMA1_29; AMA2_17	Peptidase M15	Pfam08291
AMA1_32; AMA2_19	Deoxycytidylate deaminase/MafB19-like deaminase	Pfam00383/Pfam14437
AMA1_33;AMA2_20	Thymidylate	Pfam00303
AMA1_35; AMA2_22	Phosphoribosyl-ATP pyrophosphohydrolase	Pfam01503
AMA1_47; AMA2_37	PD-(D/E)XK nuclease	Pfam12705
AMA1_53; AMA2_44	Primase C terminal 2	Pfam08707
AMA2_67	Tail component	Pfam04883
AMA2_3	Bacteriophage tail collar protein	Pfam07484
